# ESE1/AGR2 axis antagonizes TGF‐β‐induced epithelial‐mesenchymal transition in low‐grade pancreatic cancer

**DOI:** 10.1002/cam4.5397

**Published:** 2022-11-03

**Authors:** Hui‐Jing Xu, Jing Bai, Ye Tian, Xiao Feng, Ai‐Ping Chen, Jie Wang, Jin Wu, Xu‐Ru Jin, Feng Zhang, Mei‐Yu Quan, Chengshui Chen, Kwang‐youl Lee, Jin‐San Zhang

**Affiliations:** ^1^ International Collaborative Center on Growth Factor Research, and School of Pharmaceutical Sciences Wenzhou Medical University Zhejiang China; ^2^ College of Pharmacy and Research Institute of Drug Development Chonnam National University Gwangju Republic of Korea; ^3^ The Quzhou Affiliated Hospital of Wenzhou Medical University, Quzhou People's Hospital Zhejiang China; ^4^ Medical Research Center, and Department of Pulmonary and Critical Care Medicine The First Affiliated Hospital of Wenzhou Medical University Zhejiang China

**Keywords:** AGR2, epithelial‐mesenchymal transition, ESE1, pancreatic cancer, transcription factor

## Abstract

Epithelium‐specific ETS transcription factor 1 (ESE1) has been implicated in epithelial homeostasis, inflammation, as well as tumorigenesis, and cancer progression. However, numerous studies have reported contradictory roles—as an oncogene or a tumor suppressor of ESE1 in different cancers, and its function in the development and progression of pancreatic ductal adenocarcinoma (PDAC) has remained largely unexplored. Herein, we report that ESE1 was found upregulated in primary PDAC compared to normal pancreatic tissue, but high expression of ESE1 correlated to better relapse‐free survival in patients with PDAC. Interestingly, ESE1 was found to exhibit dual roles in regulation of malignant properties of PDAC cells in that its overexpression promoted cell proliferation, whereas its downregulation enhanced epithelial‐mesenchymal transition (EMT) phenotype. In the context of TGF‐β‐induced EMT, ESE1 is markedly downregulated at post‐transcriptional level, and reconstituted ESE1 expression partially reversed TGF‐β‐induced EMT marker expression. Furthermore, we identify AGR2 as a novel transcriptional target of ESE1 that participates in TGF‐β‐induced EMT in PDAC. Collectively, our findings reveal an ESE1/AGR2 axis that interacts with TGF‐β signaling to modulate EMT phenotype in PDAC.

## 1 INTRODUCTION

Pancreatic ductal adenocarcinoma (PDAC), the fourth leading cause of cancer‐associated deaths in the United States, has a 5‐year survival rate of below 10%.^1^, ^2^ Major factors that contribute to the poor prognosis of PDAC include late diagnosis, poor response to chemoradiotherapy, as well as epithelial–mesenchymal transition (EMT)‐related early invasion, and metastasis.^3^ Therefore, a better understanding of the regulatory mechanisms underlying the EMT process will facilitate the development of novel therapeutic strategies against PDAC.

Epithelium‐specific ETS transcription factor 1 (ESE1), also known as ELF3, Ert, Esx, and Jen,^4^, ^5^, ^6^, ^7^ belongs to the ETS‐domain transcription factor superfamily.^8^ As the name suggests, ESE1 is primarily expressed in epithelial‐rich tissues and regulates epithelial cell proliferation and differentiation, such as that of intestinal, lung, bladder, breast, and squamous epithelia.^6^, ^9^, ^10^, ^11^ Although ESE1 deregulation is frequently associated with cancer development and progression, its role in the pathogenesis of epithelial cancer is highly variable with regards to its impact on cell proliferation and EMT. For instance, ESE1 is reported to negatively regulate EMT in bladder and ovarian cancer cells,^12^, ^13^ whereas in non‐small cell lung cancer (NSCLC)^14^ and hepatocellular cancer (HCC),^15^ it promotes cell proliferation and EMT. Similar conflicting results have been reported in breast cancer cells. For example, ectopic expression of ESE1 decreases estrogen (E2)‐dependent MCF7 cell proliferation by inhibiting the transcriptional activity of estrogen receptor α (Erα),^16^ and plays a tumor suppressor role. In human epidermal growth factor receptor 2 (HER2)^+^ breast cancer cells, knockdown of ESE1 inhibits cell proliferation and tumor sphere formation by inhibiting HER2‐dependent signaling, therefore displaying a pro‐tumorigenic role.^17^, ^18^, ^19^ These results imply that the effects of ESE1 is dependent on the cellular context or cancer type. Moreover, although the function of ESE1 in several types of epithelial cancers has been studied, its role and mechanism in the development and progression of PDAC have remained largely unexplored.

Anterior gradient protein 2 (AGR2) is a member of the protein disulfide isomerase (PDI) family predominantly localized to the endoplasmic reticulum (ER). AGR2 expression is also mainly restricted to epithelial cells, and is associated with cancer cell proliferation, survival and tumor growth, as well as invasion and metastasis.^20^, ^21^, ^22^, ^23^ AGR2 contributes to maintain epithelial cell phenotype, inhibits EMT induction, and enhances the rate of adhesion to plastic substratum, thus playing an undeniable role in tumor development and progression.^24^, ^25^ However, the transcriptional regulation of AGR2 in the setting of cancer cell proliferation, migration, and invasion has not yet been fully elucidated.

Here, we investigated whether ESE1 contributes to TGF‐β‐induced EMT in PDAC cells. In addition, we identified potential targets of ESE1 mediating such activity. Our results indicated that ESE1 plays an important role in inhibiting TGF‐β‐induced EMT in PDAC cells via the transcriptional activation of AGR2.

## 2 MATERIALS AND METHODS

### 2.1 Cell culture and selection of stable cell clones

HEK293T, HeLa, and PDAC lines L3.6, PaTu 8988 t, PANC‐1, MIA PaCa‐2, CFPAC‐1, and HPNE, an immortalized human pancreatic epithelial cell line, were maintained and transfected as previously described.^26^ For stable knockdown of the *ESE1* mRNA, lentiviral particles generated from pLKO‐based lentiviral vector carrying shRNAs targeting human *ESE1* or scramble control were used for cell transduction. The cells were selected in puromycin‐containing media as previously described.^26^ The shRNA sequences can be found in Table S1. For reconstituted overexpression of the *ESE1* gene, shESE1 L3.6 cells were transfected with lentiviral particles carrying the pLenti6.3‐Flag‐ESE1 cDNA. The infected cells were selected with blasticidin‐containing media (Sigma‐Aldrich, USA). Pooled antibiotic‐resistant cell clones were used for indicated experiments.

### 2.2 Quantitative RT‐PCR (qPCR)

Total RNA was extracted using TRIzol reagent (Invitrogen, USA) and reverse transcribed into cDNA using PrimeScript™ RT reagent Kit (TaKaRa, Japan). The cDNA products were used as templates for qPCR using iTaq™ Universal SYBR® Green Supermix (Bio‐rad, USA) on a Roche Light Cycler 96 System Real‐Time System. All the genes were normalized by *GAPDH*. The primer sequences are listed in the Table S1.

### 2.3 Western blotting

The cells were lysed with lysis buffer (Beyotime, China) freshly supplemented with phosphatase inhibitors and phenylmethanesulfonyl fluoride (Beyotime, China). The samples were fractionated by SDS‐PAGE gels and transferred to microporous polyvinylidene difluoride (PVDF) membranes (Roche, Swiss). The membranes were blocked with 5% skim milk for 1 h and incubated overnight at 4°C with primary antibodies including rabbit polyclonal anti‐AGR2^27^; ESE1 (Santa Cruz Biotechnology, USA); E‐cadherin (CST#3195, USA), N‐cadherin (CST#13116, USA), and vimentin (CST#5741, USA); GAPDH (10494‐1‐AP, Proteintech, USA). The membranes were then washed and probed with secondary antibodies: goat anti‐mouse IgG (H + L)‐HRP or goat anti‐rabbit IgG (H + L)‐HRP (Bioworld, USA) before detection with the enhanced chemiluminescence (Thermo Fisher Scientific, USA) and imaged using the ChemiDoc XRS + System (Bio‐Rad, USA).

### 2.4 Cell proliferation and migration assays

Cell proliferation assays were performed as directed by CCK‐8 kit (Dojindo, Japan). Briefly, single cell suspensions were seeded in 96‐well plates at a density of 5000 cells/well and cultured for 0, 24, 48, and 72 h, respectively, before adding 10% of CCK‐8 solution and incubated for another 2 h. The absorbance values were measured at 450 nm by microplate reader (BioTek, USA). Scratch healing assays were performed as previously described.^28^ Cells were cultured overnight to reach 90% confluency in a 6‐well plate before scratching with a 20‐μl plastic pipette tip. The cells that migrated into the wounded areas were imaged with the Microscope (Leica, Germany). Cell migration rate was measured as follows: (original wound width ‐ new wound width) / original wound width× 100%. For TGF‐β (MCE, USA) treatment, serum‐free DMEM supplemented with TGF‐β (15 ng/ml) was added to replace the serum‐free DMEM without TGF‐β. Tracked cell migration were performed using Operetta CLS system (PerkinElmer, USA). After overnight culture, cells were administered with TGF‐β (15 ng/ml) for 3 days and were tracked for 10 h with the Operetta CLS system. Cell distribution, migration speed, and tracks were analyzed with Harmony software.

### 2.5 Luciferase reporter assays

*AGR2* promoter DNA sequence (−1506 to −99 relative to the translation start site) was cloned into pGL3‐basic vector (pGL3‐AGR2). The cDNA sequences encoding ESE3, ESE1, and its truncation mutants were cloned into pM vector (Clontech, USA) in frame with N‐terminal GAL4‐DBD (GAL4‐ESE1 or GAL4‐ESE3). The pRL‐TK renilla vector and pM‐GAL4 reporter plasmid have previously been described.^29^ For AGR2 promoter luciferase reporter assay, pGL3‐AGR2 reporter was co‐transfected with ESE1 or SPDEF cDNA expression plasmid with the pRL‐TK vector as internal control for transfection efficiency; to determine the transcriptional regulatory activity of ESE1 and ESE3, GAL4‐ESE1(and its truncation mutants as indicated), GAL4‐ESE3 plasmids were co‐transfected with pGL3 firefly reporter plasmid carrying five tandem GAL4 DNA binding sites (GAL4‐luc) as previously described.^29^ Transfections were carried out following standard procedures, and the dual‐luciferase assays were performed with LucPair™ Duo‐Luciferase Assay Kit (GeneCopoeia, USA). To determine the effect of TGF‐β on ESE1 transcriptional activity, cells were treated with 15 ng/mL of TGF‐β (MCE, USA) for 3 days before harvesting for the above assays.

### 2.6 Immunofluorescent staining and imaging

Immunofluorescence (IF) imaging was used to determine the subcellular localization of endogenous ESE1 as recently described.^28^ To determine the localization of overexpressed ESE1, ESE1‐EGFP, or ESE1‐Flag expression plasmids were transfected to HeLa cells using Lipo2000 regents. Cells were fixed and stained at 24 h after transfection. Images were captured using the Leica 2500 Microscope System (Leica, Germany).

### 2.7 Gene expression profiling and survival analysis

We downloaded the gene expression profiles from GSE16515 and GSE16950 databases. The expression of ESE1 was visualized using the GraphPad Prism 7 and the GEPIA database. The relapse‐free survival (RFS) and overall survival (OS) analyses for ESE1 or AGR2 were performed through the KM‐plotter.^30^

### 2.8 Functional annotation and transcription factor interactome analysis

Functional annotation analysis was performed using the OmicStudio tools available at https://www.omicstudio.cn/tool.

### 2.9 Gene set enrichment analysis

The GSEA enrichment of KEGG pathways was performed at https://www.omicstudio.cn/tool. The following list of genes were used for GSEA: differentially expressed genes (DEGs) in L3.6‐shCtrl cells versus L3.6‐shESE1.

### 2.10 Statistical analysis

Statistical analysis was performed using the GraphPad Prism software (version 7; GraphPad Software, Inc.). Multiple comparisons were performed between the groups using one‐way analysis of variance (ANOVA), followed by multiple comparison tests using the GraphPad Prism software. Data are expressed as mean ± standard deviation. *p*‐value <0.05 indicated a significant difference.

## 3 RESULTS

### 3.1 ESE1 is overexpressed in human PDAC

**FIGURE 1:**
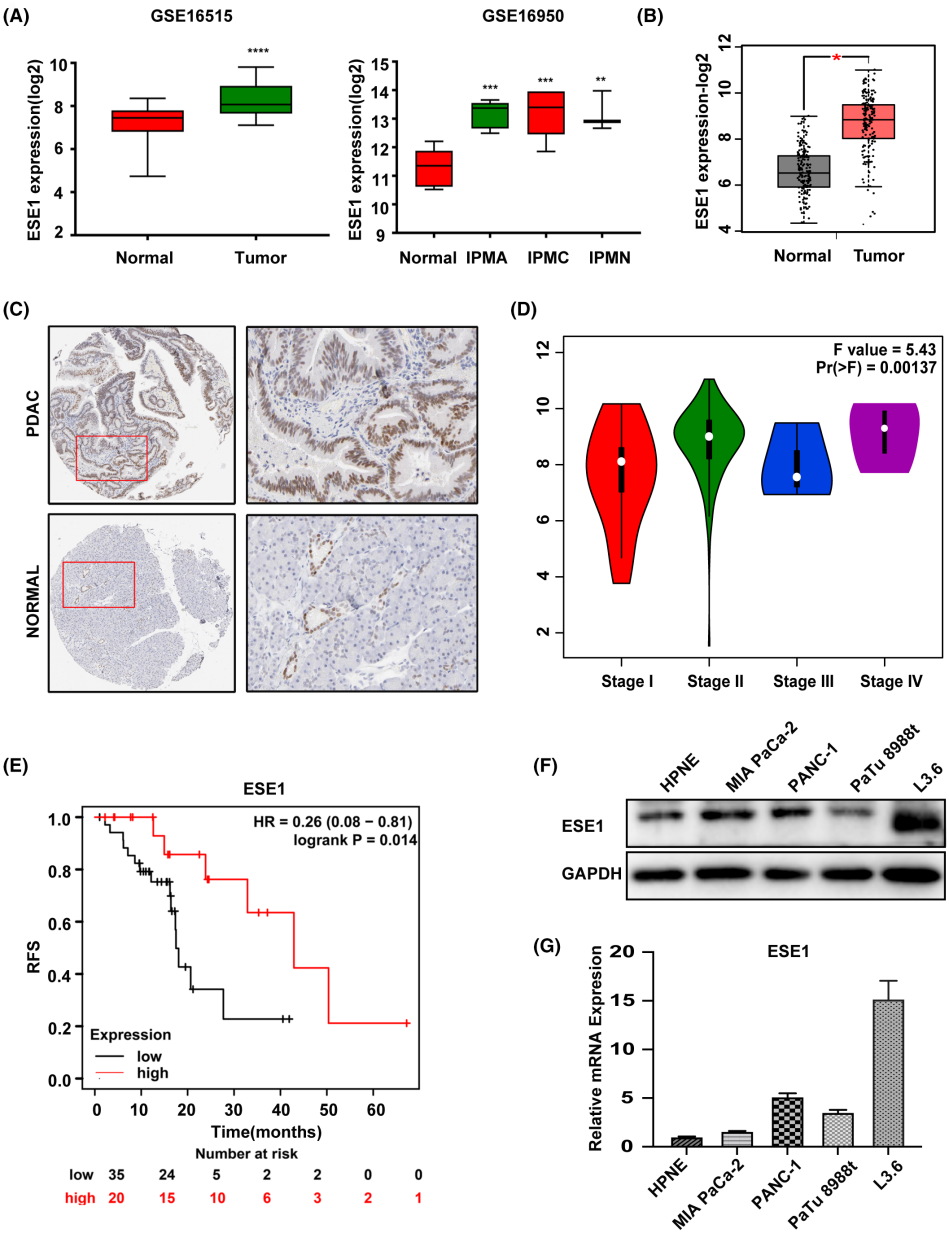
ESE1 is overexpressed in human PDAC. (A) The expression level of ESE1 in the GEO datasets. (B) The expression level of ESE1 in primary PDAC tissues and matched normal tissue based on TCGA dataset. (C) IHC of ESE1 based on the data from Protein Atlas database. (D) Statistical analysis of ESE1 expression with tumor progression (clinical stages). (E) The correlation between ESE1 expression with RFS in stage‐2 PDAC patients based on the data obtained and analyzed from Kaplan–Meier Plotter database. ESE1 expression in four PDAC cell lines by western blotting (F) and qPCR (G). HR, Hazard Ratio; IPMA, intraductal papillary mucinous adenoma; IPMC, intraductal papillary‐mucinous carcinoma; IPMN, intraductal papillary mucinous neoplasm; PDAC, pancreatic ductal adenocarcinoma.

We found *ESE1* highly overexpressed in various human tumors including colorectal adenocarcinoma and PDAC compared to maching normal tissues based on GEPIA (Figure S1A). We further analyzed two Gene Expression Omnibus (GEO) datasets (GSE16515 and GSE16950)^31^, ^32^ and found significantly higher ESE1 expression in PDAC or precancerous lesions including IPMA, IPMC, and IPMN than in matching normal tissues (Figure 1A). In addition, consistent results were obtained in the TCGA database with GEPIA (Figure 1B). The immunohistochemistry (IHC) data from the Protein Atlas database (https://www.proteinatlas.org/ENSG00000163435) showed a higher number of ESE1‐positive cells in PDAC than in normal pancreatic tissues (Figure 1C). Interestingly, ESE1 expression decreased with tumor stage, and low ESE1 expression was correlated with shorter RFS, but better OS (Figure 1D,E, Figure S1B). These results reflected a dichotomy of ESE1 association with pancreatic cancer progression and prognosis. The relative expression of ESE1 was further determined in a panel of PDAC cell lines with L3.6 showing the highest levels of both mRNA and protein expression (Figure 1F,G).

### 3.2 Differential impact of ESE1 on PDAC cell proliferation and EMT phenotype

**FIGURE 2:**
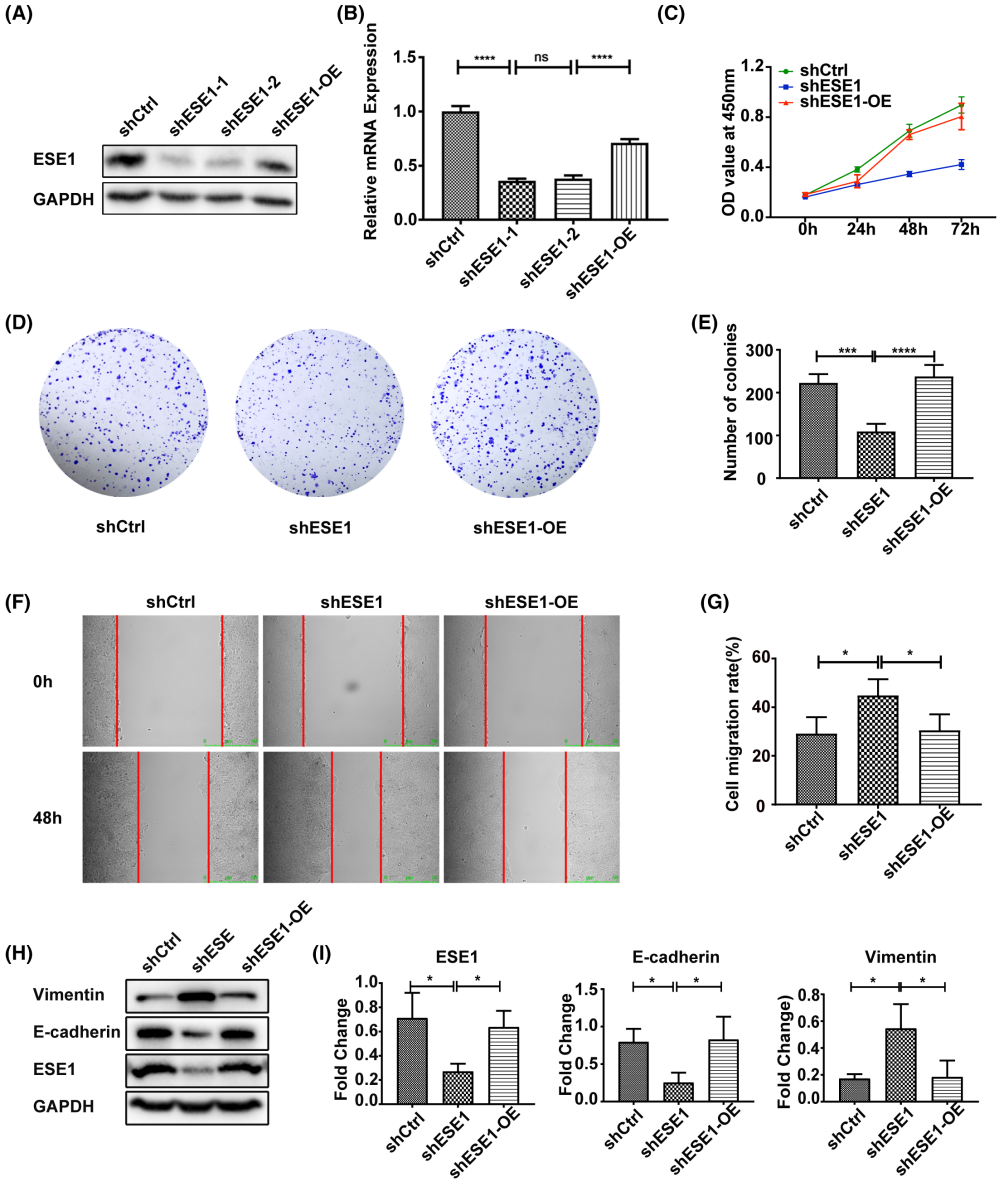
Differential impact of ESE1 on PDAC cell proliferation and EMT phenotype. (A, B) Validation of ESE1 knockdown in L3.6 cells by Western blotting and qRT‐PCR. (C) The effect of ESE1 on L3.6 cell proliferation was determined with CCK‐8 assay. (D) Representative images of colony formation assay in L3.6 cells transfected with scrambled control (shCtrl), ESE1 specific shRNA (shESE1), and reconstituted ESE1 expression in ESE1 knockdown cells (shESE1‐OE). (E) Statistical analysis of colony formation assay result. (F) Representative images of wound‐healing assay in L3.6 cells from shCtrl, shESE1, and shESE1‐OE groups, respectively. (G) Statistical analysis of the wound‐healing assay results shown in (F). (H) Immunoblot detection of EMT marker expression in L3.6‐shESE1 and shESE1‐OE cells. (I) Quantitative analysis of the target protein bands using ImageJ software. Data are presented as means ± SEM (*n* = 3 per group). *p < 0.05, ***p < 0.001, ****p < 0.0001. Scale bars: 500 μm (F).

To explore the function of ESE1 expression in PDAC cells, we selected L3.6 with high ESE1 expression for subsequent experiments. ESE1 stable knockdown cell lines were obtained by transducing cells with pLKO‐based lentiviral shRNA targeting the human ESE1 mRNA (with a non‐targeting shRNA as the control) (Figure 2A,B). Cell proliferation ability of L3.6 cells was significantly reduced by shRNA‐mediated suppression of endogenous ESE1 compared with scramble control, whereas reconstituted expression of ESE1 mostly restored cell proliferation potential compared with the shESE1 group in L3.6 cells (Figure 2C). Similar results were obtained with colony formation assays (Figure 2D,E). Contrary to its inhibition of cell proliferation and colony formation, the shESE1 stable cells exhibited higher migration capabilities (Figure 2F,G), whereas reconstituted overexpression of ESE1 largely reversed this enhanced migration ability compared with the ESE1 knockdown control. Therefore, ESE1 contributed to promote the proliferation, but inhibited the cell migration ability of L3.6 cells. Next, we investigated the potential influence of ESE1 on EMT process and found ESE1 knockdown markedly increased the expression level of the mesenchymal marker vimentin and decreased that of E‐cadherin, whereas the changes in the expression levels of both markers were largely reversed upon reconstituted expression of ESE1 (Figure 2H,I). Considering that ESE1 showed high expression in low‐grade and low‐to‐undetectable expression in high‐grade PDACs,^33^ we selected another well differentiation pancreatic cell line CFPAC‐1 to explore the function of ESE1 expression.^34^ Similar to the findings in L3.6 cells, ESE1 suppression also inhibited CFPAC‐1 proliferation (Figure S2A‐C), while promoting its EMT phenotype (Figure S2D). These results suggest that ESE1 is a critical component of the EMT machinery in these PDAC cells, leading to the hypothesis that downregulation of ESE1 promoted a EMT phenotype.

### 3.3 Knockdown of ESE1 expression affects the TGF‐β network in PDAC cells

**FIGURE 3:**
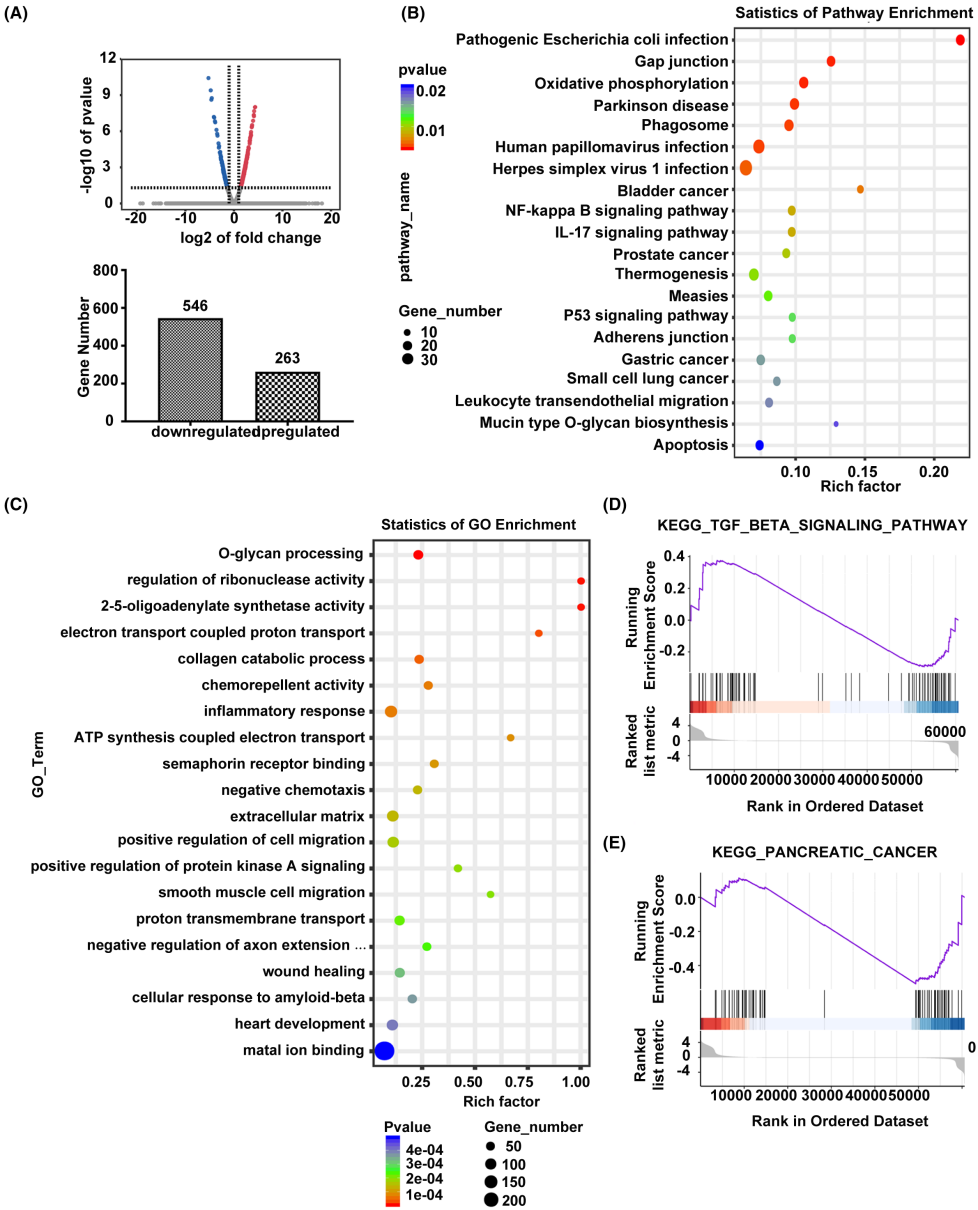
Knockdown of ESE1 expression affects the TGF‐β network in PDAC cells. RNA‐seq analysis was performed in L3.6‐shCtrl versus L3.6‐shESE1 cells to determine the DEGs. (A) Volcano map of DEGs. The blue dots represented down‐regulated genes while red dots represented up‐regulated genes with the number of down‐ and up‐regulated genes shown blow. (B) Bubble map of DEGs in L3.6‐shCtrl versus L3.6‐shESE1 cells based on KEGG pathway analyses. (C) Bubble map of GO pathway analyses on DEGs in L3.6‐shCtrl versus L3.6‐shESE1 cells. GSEA analysis based on DEGs in L3.6‐shCtrl versus L3.6‐shESE1 cells to compare the changes related to TGF‐β signaling pathway (D) and pancreatic cancer (E) using the KEGG gene sets.

To identify the potential transcriptional targets of ESE1, we next performed RNA sequencing analysis in L3.6 cells with ESE1 knockdown versus non‐targeting control (GSE 206999). A total of 546 downregulated genes and 263 upregulated genes were found to be associated with the suppression of ESE1 (*p* < 0.01; log2 fold change >1; Figure 3A, GSE 206999). The Kyoto Encyclopedia of Genes and Genomes (KEGG) pathway enrichment analysis was applied for pathway annotations and showed that DEGs were enriched in cancer signaling pathways, including pathways related to bladder cancer, prostate cancer, gastric cancer, and small cell lung cancer (Figure 3B). ESE1‐induced DEGs were further analyzed by functional annotations in Gene Ontology (GO) term, and the results revealed that genes associated with carcinogenic pathways are enriched, including inflammatory response, extracellular matrix, positive regulation of cell migration, wound healing (Figure 3C). Because of the intricate association between ESE1 and cell migration, and the enrichment of wound healing and DEGs in the cancer pathways, we hypothesized that ESE1 could be a target of EMT‐related pathways in PDAC cells, such as TGF‐β. The KEGG pathway correlated with the DEGs was then studied using gene‐set enrichment analysis (GSEA) to further investigate the biological functions of DEGs (https://www.omicstudio.cn/tool). Indeed, the DEGs were found to be enriched in the TGF‐β signaling pathway and also related to pancreatic cancer (Figure 3D,E). The GSEA of the microarray data and the fact that TGF‐β signaling is a predominant promoter of EMT and PDAC progression^35^, ^36^ prompted us to examine the role of ESE1 in TGF‐β‐induced EMT process in pancreatic cancer.

### 3.4 TGF‐β‐induced EMT is associated with ESE1 post‐transcriptional downregulation

**FIGURE 4:**
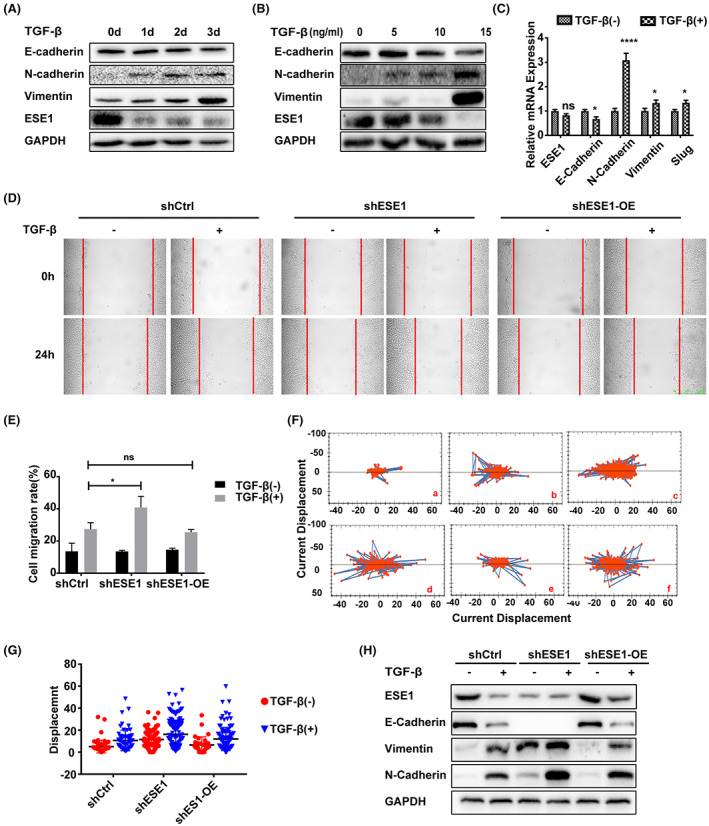
TGF‐β‐induced EMT is associated with ESE1 post‐translational downregulation. Immunoblot analysis to determine ESE1 and EMT‐related marker expression in L3.6 cells treated with TGF‐β (15 ng/mL) for indicated time (A) and doses (B). (C) The mRNA expression of ESE1 and EMT markers was determined by qRT‐PCR analysis in L3.6 cells treated with and without TGF‐β for 3 days. (D) Representative images of wound‐healing assay from shCtrl, shESE1 and shESE1‐OE groups with and without TGF‐β treatment. (E) Quantitative analysis of the scratch‐healing assay result by measuring the cell migration rate. (F) Cell track analysis in shCtrl, shESE1, and shESE1‐OE cells without (a, c, and e) and with TGF‐β treatment (b, d, and f) by Operetta CLS. (G) Quantitative analysis of the cell displacement with Harmony software. (H) Immunoblot analysis of ESE1 and EMT marker protein expression in L3.6 cells with and without TGF‐β treatment. Data are presented as means ± SEM (*n* = 3 per group). *p < 0.05, ****p < 0.0001. Scale bars:500 μm (D).

EMT is a critical biological process that contributes to the tumor cell migration and invasion.^37^ We investigated whether ESE1 regulated the EMT process in L3.6 cells. We first analyzed the expression levels of EMT markers, including E‐cadherin, N‐cadherin, and vimentin. The results showed that TGF‐β increased the expression levels of the mesenchymal markers N‐cadherin and vimentin, while decreasing the expression level of the epithelial marker E‐cadherin. Furthermore, the protein expression of ESE1 was significantly reduced after the treatment with TGF‐β (15 ng/mL) on the first day accompanied by induced N‐cadherin expression, whereas the expression of vimentin started increasing on the second day and becoming more apparent on the third day (Figure 4A). Similarly, cells treated with different concentrations of TGF‐β showed reduced expression of ESE1 before increased expression of vimentin (Figure 4B). These findings suggested that TGF‐β contributed to downregulate ESE1 protein expression in L3.6 cells. However, qRT‐PCR analysis revealed no significant difference at the mRNA levels of ESE1 in L3.6 cells following TGF‐β treatment suggesting that TGF‐β did not affect the transcription of ESE1 (Figure 4C). The results also showed increased expression of the mesenchymal marker vimentin and decreased expression of epithelial marker E‐cadherin in CFPAC‐1 cells with TGF‐β treatment. Furthermore, the protein expression of ESE1 was significantly reduced after the treatment with TGF‐β, which was consistent with above findings on TGF‐β‐treated L3.6 cells (Figure S2E).

To confirm that ESE1 is a key component of TGF‐β‐induced EMT, we investigated whether altered ESE1 expression could influence TGF‐β‐induced EMT in L3.6 cells. Scratch wound healing assays were carried out on cells that had been pre‐treated with TGF‐β for 72 h to initiate the EMT process. The results demonstrated that the migration ability of shESE1 cells was dramatically increased following TGF‐β treatment, whereas rescued overexpression of ESE1 (shESE‐OE) attenuated this effect (Figure 4D,E). These results were further validated using cell‐tracking assays performed using the Operetta CLS high content analysis system (Figure 4F,G). Compared with the shCtrl group, the shESE1 group demonstrated significantly increased displacement. Compared with the shESE1 group, the shESE1‐OE group showed significantly reduced migration. Similarly, the expression of vimentin and N‐cadherin was significantly increased in *ESE1* knockdown cells treated with TGF‐β, whereas overexpression of *ESE1* relieved the increased levels of vimentin and N‐cadherin in shESE1‐OE cells (Figure 4H). Immunofluorescent staining revealed mesenchymal‐like morphologies of shESE1 cells treated with TGF‐β in L3.6 cells (Figure S3). Similarly, compared with the shCtrl group, the shESE1 group demonstrated significantly increased displacement in CFPAC‐1 cells (Figure S2F). These data indicate that TGF‐β induces EMT in PDAC cells by downregulating ESE1.

### 3.5 Downregulation of ESE1 and AGR2 highly correlates to EMT phenotype

**FIGURE 5:**
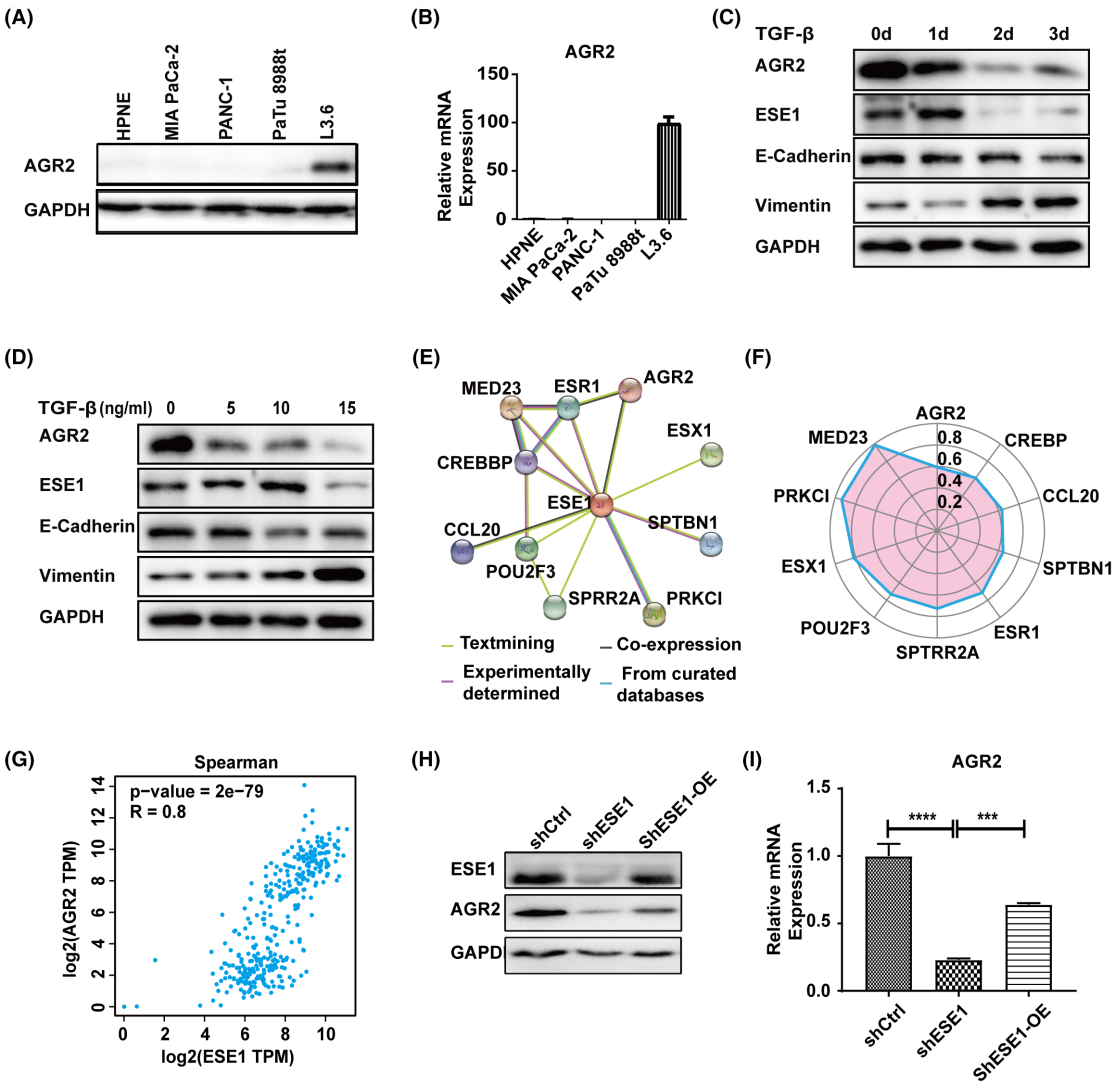
Downregulation of ESE1 and AGR2 highly correlates to EMT phenotype. The relative protein and mRNA expression of AGR2 in PDAC cell lines and immortalized normal pancreatic epithelial cell line (HPNE) by Western blotting (A) and qRT‐PCR (B). Immunoblot analysis to determine AGR2, ESE1 and EMT‐related marker expression in L3.6 cells treated with TGF‐β for indicated time (C) and doses (D). (E) Network analysis of ESE1 correlation by STRING database. (F) Analysis of the ESE1 interaction gene score. (G) The correlation analysis between AGR2 and ESE1 based on Spearman correlation analysis of GEPIA database. The protein (H) and mRNA (I) expression of AGR2 in L3.6‐shCtrl, L3.6‐shESE1, and L3.6‐shESE1‐OE groups. Data are presented as means ± SEM (*n* = 3 per group). ***p < 0.001, ****p < 0.0001.

Overexpression of *AGR2* was associated with advanced clinical stage, advanced tumor, but a better prognosis based on the TCGA database and KM‐plotter database in PDAC (Figure S4A‐C), suggesting a complex effect of AGR2 on cancer growth and survival resembling that of ESE1. Similar to ESE1, the mRNA and protein expression of AGR2 were found to be higher in L3.6 than the others tested (Figure 1F, Figure 5A,B). Consistent with the previous reports that TGF‐β downregulates the expression of *AGR2* in a cell context‐dependent manner,^38^ we found that *AGR2* was significantly downregulated during TGF‐β‐induced EMT in L3.6 and CFPAC‐1 cells (Figure 5C,D, Figure S2E). Therefore, we hypothesized that AGR2 may participate in ESE1‐mediated inhibition of EMT. To test this hypothesis, we performed protein–protein interaction (PPI) network functional enrichment analysis for ESE1 protein using the Search Tool for the Retrieval of Interacting Genes/Proteins (STRING) database (https://version11.string‐db.org/cgi/network.pl?taskId=VZEEHh0QUtmm). The analysis showed a positive relationship between ESE1 and AGR2, implying that ESE1 interacts with AGR2 in PDAC (Figure 5 E,F). Bioinformatics analysis of transcriptome sequencing data showed that compared with shCtrl, the expression of AGR2 was significantly downregulated in shESE1 cells (GSE 206999). Similar to the effect of *ESE1* knockdown, shRNA‐mediated suppression of *AGR2*, as demonstrated in Figure S4D,E, also reduced the colony formation, but promoted the migration of L3.6 cells, further revealing its resemblance of ESE1 in differential regulation of PDAC cell proliferation and migration (Figure S4F,G). Moreover, similar to the effect of *ESE1* knockdown on EMT markers, we found AGR2 knockdown markedly increased the expression level of the mesenchymal marker vimentin and decreased that of E‐cadherin in shAGR2 L3.6 cells (Figure S4H). The correlation between AGR2 and ESE1 was further supported by Spearman's correlation coefficient analysis (http://gepia.cancer‐pku.cn/detail.php?clicktag=correlation) (Figure 5G). We next determined the relationship between endogenous ESE1 and AGR2 in L3.6 cells. The results showed that the levels of AGR2 mRNA and protein were significantly reduced upon suppression of endogenous ESE1 (Figure 5H,I, Figure S5A). Similarly, the levels of AGR2 mRNA (data not shown) and protein were significantly reduced upon suppression of endogenous ESE1 in CFPAC‐1 cells (Figure S2D). These results suggest AGR2 a potential transcriptional target of ESE1.

### 3.6 TGF‐β inhibits ESE1‐mediated transactivation of AGR2 promoter

**FIGURE 6:**
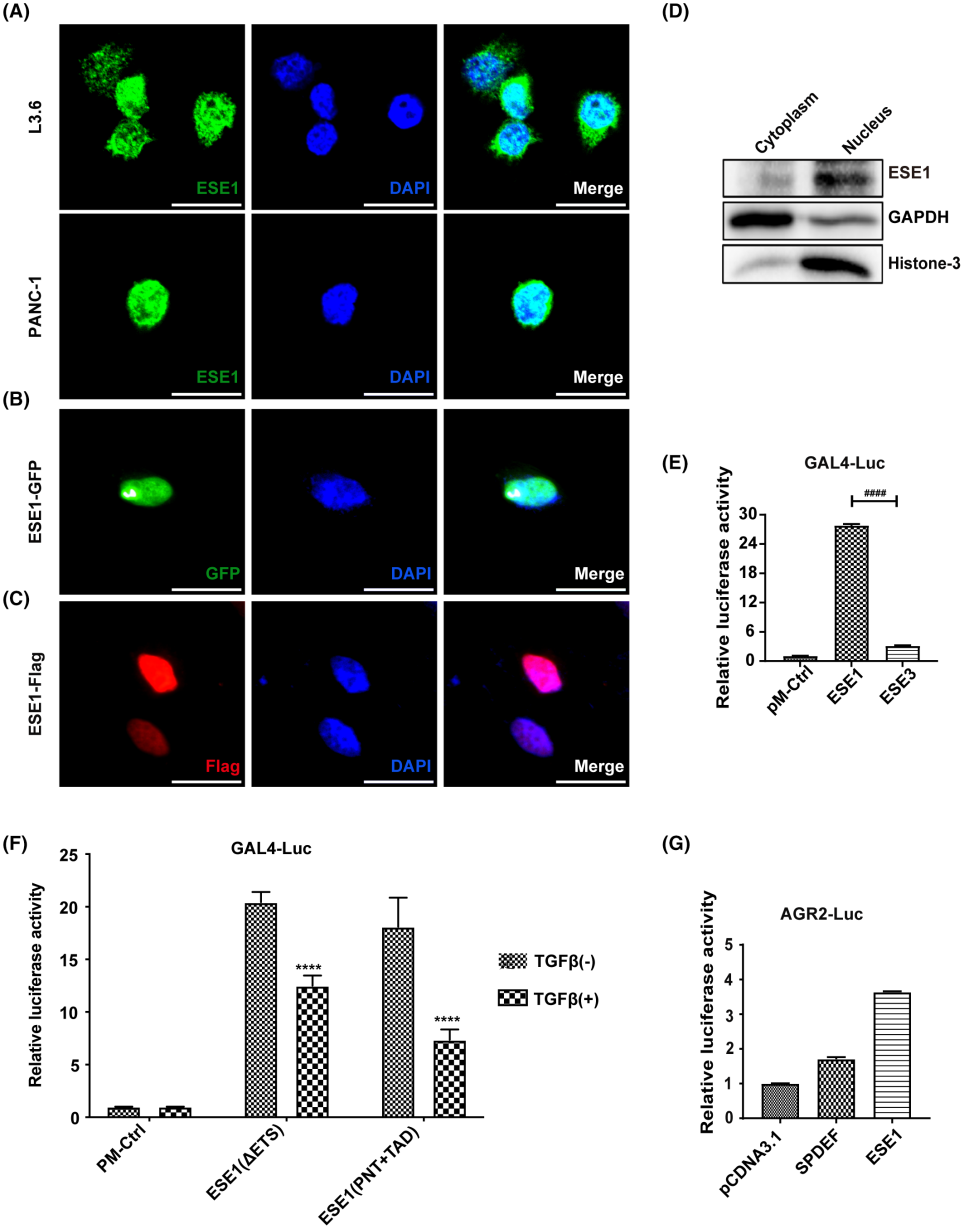
TGF‐β inhibits ESE1‐mediated transactivation of AGR2 promoter. (A) Detection of endogenous ESE1 expression and localization in L3.6 and PANC‐1 cells by IF. ESE1(green), nuclei (DAPI). HeLa cells were transfected with ESE1‐GFP (B) or ESE1‐flag expression plasmids (C) to reveal their predominant nuclear localization. (D) ESE1 protein expression in L3.6 cells was assessed by immunoblot analysis of cytoplasmic and nuclear fractions. (E) The ability of GAL4 DBD‐ESE1 and GAL4 DBD‐ESE3 fusion proteins to activate the GAL4‐Luc reporter in L3.6 cells. (F) GAL4‐Luc reporter assay to determine the activity of GAL4 DBD‐ESE1 fusion proteins in L3.6 cells with or without TGF‐β treatment. (G) Luciferase reporter assay to determine the transactivation of AGR2 promoter by ESE1 relative to the positive control SPDEF. Data are presented as means ± SEM (*n* = 3 per group). ****p < 0.0001 compared with control, ^####^ *p* < 0.0001 intergroup comparison. Scale bars: 20 μm. PNT, TAD, and ETS indicate Sterile alpha motif/Point domain, transactivation domain, and ETS DNA binding domain, respectively. AGR2‐Luc, AGR2 luciferase reporter.

To elucidate whether ESE1 functions as a cytoplasmic regulator like previously reported in breast cancer or as a nuclear transcriptional factor like most other ETS proteins, we examined the expression of endogenous ESE1 localization by IF and immunoblot analysis of the nuclear and cytoplasmic extracts in L3.6, PANC‐1, and CFPAC‐1. We found ESE1 predominantly localized in the nuclear (Figure 6A,D, Figure S5B). Similar results were observed in HeLa cells with exogenously overexpressed ESE1 either with a C‐terminally fused GFP or an N‐terminal flag tag (Figure 6B,C). GAL4‐based heterologous reporter assay detected much stronger activation of luciferase reporter by ESE1, compared to its close homolog ESE3 (Figure 6E). Further analysis of ESE1 truncation mutants indicated that N‐terminal half of ESE1 containing the pointed and the transcription activation domains (PNT + TAD) were able to robustly transactivate the GAL4‐Luciferase reporter in all tested PDAC lines including BxPC3, L3.6, PANC‐1, and Patu8988T cells (Figure S6A,B). Importantly, TGF‐β treatment significantly reduced ESE1‐mediated transactivation of the reporter (Figure 6F). Given the crucial role of ESE1 in maintaining the expression of the endogenous AGR2 (Figure 5H, Figure S5A), we next examined whether ESE1 could directly activate AGR2 promoter. ESE1‐flag expression vector was co‐transfected with pGL3‐AGR2 luciferase reporter constructs in HeLa cells with Flag empty vectors as negative control, and the SAM pointed domain‐containing ETS transcription factor (*SPDEF*), a gene reported to transcriptionally activate AGR2,^39^, ^40^ as the positive control. ESE1 was found to more robustly increased the activity of AGR2 promoter than *SPDEF* (Figure 6G). These results indicated that ESE1 is a strong activator of AGR2 promoter transcription, and its activity is regulated by TGF‐β signaling.

## 4 DISCUSSION

Many studies reported contradictory roles of ESE1 as an oncogene or a tumor suppressor depending on the cancer types or different malignant properties, highlighting a complexed nature and mechanism of its action.^13^, ^41^, ^42^, ^43^ The current work illustrated that ESE1 exhibits dual roles in regulation of malignant phenotypes in that its overexpression promoted cell proliferation, whereas its downregulation enhanced EMT phenotype in the L3.6 and CFPAC‐1 PDAC cells. Our data establish ESE1 as a negative regulator of TGF‐β‐induced EMT in PDAC. We further identify AGR2 as a novel transcriptional target of ESE1, that participates in TGF‐β‐induced EMT. These findings provide mechanistic insight into the differential roles of ESE1 in regulating different cell malignant properties and offer a potential explanation for its seemingly contradicting impact on RFS and OS in PDAC patients, as well as some early conflicting results reported in different types of cancer. Given the growing interest in ETS‐directed therapies, such as with small molecule inhibitors or siRNA,^44^, ^45^ our results raise necessary precautions for applying this type of approach, and a better mechanistic understanding will facilitate design of drugs with desired inhibition of tumor growth, while eliminating the potential adverse effects on promoting EMT and metastasis.

TGF‐β signaling is a predominant driver of EMT, a key initial step of cancer progression in various epithelial cancer including PDAC.^46^ Previous studies have reported ESE1 as a transcriptional activator of TGF receptor type II (TGFβR‐II). ESE1 directly binds TGFβR‐II promoter and robustly increases its expression in breast cancer and the mouse embryonal carcinoma cells.^47^ Genetic ablation of mouse ESE1 gene was associated with highly reduced TGFβR‐II expression.^48^ Significantly, the developmental defect can be partially rescued by transgenic expression of Tgf‐βRII in ESE1(−/−) background. Together with the crystal structure of a mouse Elf3 complexed with mouse TGFβR‐II promoter DNA,^49^ these studies established ESE1 as a bona fide and crucial upstream regulator of TGF‐β signaling both in the embryonic development and the cancer cells. The current identification of ESE1 as a downstream target of TGF signaling reveals a ESE1‐mediated feed‐back regulatory mechanism during TGF‐β‐induced EMT in PDAC, and perhaps also in other cancer. In contrast to the direct transcriptional activation of Tgf‐βRII by ESE1, we found that the expression of ESE1 protein, but not its mRNA, is markedly down regulated by TGF‐β, suggesting that decreased ESE1 expression following TGF‐β‐induced EMT is mainly a post‐transcriptional event, but the underlying mechanism remains to be further investigated.

Of note, ESE1 contains a serine‐ and aspartic rich (SAR) motif and an HMG‐like AT‐hook domain, which have been reported to confer ESE1 unique cytoplasmic localization and additional functional properties in breast cancer.^50^, ^51^ The SAR domain is found both necessary and sufficient to maintain a morphological EMT and metastatic phenotype, whereas nuclear localized ESE1 protein contributes to maintain the transformed phenotype of breast cancer cells.^52^ A monopartite nuclear localization signal (NLS1) in the AT‐hook and a three lysine residues (NLS2) within the ETS domain have been mapped.^53^, ^54^ Additionally, two CRM1‐dependent nuclear export signals(NES) located within the pointed domain and the ETS domain, respectively, have also been found.^53^ Therefore, these NLS/NES may work together to regulate cytoplasmic‐nuclear shuttling of ESE1. However, unlike those reported in the breast cancer cells, we observed predominantly nuclear localized ESE1 of both endogenous protein and transfected full‐length protein expression plasmid in PDAC cells. We suggest that cytoplasmic‐nuclear shuttling represents a key knot governing ESE1's dichotomous activities toward promoting cell proliferation and suppression of EMT that may be cancer cell type dependent and subject to regulation by additional signaling pathways, such as TGF‐β as we demonstrated. The precise control mechanism of ESE1 subcellular localization and its functional properties should be a key topic for future research.

Despite of its role as a crucial guardian of the epithelial state^41^ and a negative regulator of EMT in cancer,^12^, ^13^ the signaling pathways governing the activity and the transcriptional targets of ESE1 in mediating its cellular effects remain largely elusive. The current work identifies AGR2 as a novel and functional ESE1 transcriptionally activated target gene in PDAC. Interestingly, AGR2 has previously been identified as a TGF‐β down‐regulated gene with an essential role in governing MUC1 expression, and in facilitating mPanIN initiation and progression to PDAC.^55^ Reduced AGR2 expression in human PDAC patients correlates to EMT phenotype, aggressive histological grade and adverse outcome highly resembling that of ESE1.^56^ Similarly, in human lung adenocarcinoma, TGF‐β‐induced EMT leads to dramatic down regulation of AGR2, whereas forced overexpression of AGR2 largely reversed the TGF‐β‐induced EMT‐phenotype.^38^ An independent study further show that TGF‐β inhibits ESE‐1 expression in the same cell lines, suggesting that AGR2 downregulation is mediated, at least in part, by ESE1.^57^ Our data together with the published observations suggest ESE1/AGR2 axis serves to maintain epithelial cell identity, which is antagonized by TGF‐β signaling in cancer cells to undergo EMT.

Interestingly, both ESE1 and AGR2 are linked to ZEB1/2 regulation, a key EMT driver downstream of TGF‐β. AGR2 is identified as a direct target of *ZEB1* transcriptional repression, and that AGR2 promotes *ZEB1* mRNA degradation therefore forming a double negative feedback loop in the EMT and metastasis process. The balance between AGR2 and ZEB1 is suggested to govern the aggressiveness and invasive phenotype of tumor cells.^24^ On the contrary, *ZEB1/2* and *Snail* have also been identified as ESE1 transcriptional targets of repression in biliary tract and colorectal cancer.^58^, ^59^ In breast cancer cells, ETS1 and ESE1 reciprocally regulate the expression of *ZEB1/2*, and the net outcome is mainly dictated by ERK1/2 activity, which positively impacts on ETS1 transactivation of *ZEB1/2*, while it inhibits their transcriptional repression by ESE1.^60^ The current results together with the published work reveal an intricate regulatory network between TGF‐β signaling and ESE1/AGR2 axis that function together with other EMT drivers such ZEBs to govern the normal epithelial homeostasis or EMT phenotype in cancer cells.

## AUTHOR CONTRIBUTIONS

**Hui‐Jing Xu:** Conceptualization (lead); data curation (lead); investigation (lead); methodology (lead); writing – original draft (lead). **Jing Bai:** Conceptualization (lead); data curation (lead); investigation (lead); methodology (lead). **Ye Tian:** Data curation (equal); investigation (equal); methodology (equal). **Xiao Feng:** Data curation (equal); investigation (equal); methodology (equal). **Ai‐Ping Chen:** Data curation (equal); investigation (equal); methodology (equal). **Jie Wang:** Data curation (equal); investigation (equal); methodology (equal). **Jin Wu:** Data curation (equal); investigation (equal); methodology (equal). **Xu‐Ru Jin:** Investigation (equal); methodology (equal). **Feng Zhang:** Data curation (equal); investigation (equal); methodology (equal). **Mei‐Yu Quan:** Data curation (equal); investigation (equal); methodology (equal). **Chengshui Chen:** Conceptualization (equal); funding acquisition (equal); resources (equal); supervision (equal). **Kwang Yeol Lee:** Conceptualization (equal); funding acquisition (equal); resources (equal). **Jin‐San Zhang:** Conceptualization (lead); data curation (lead); funding acquisition (lead); investigation (lead); methodology (lead); resources (lead); supervision (lead); writing – review and editing (lead).

## CONFLICT OF INTEREST

The authors declare that they have no competing associated with the manuscript.

## ETHICS STATEMENT

This study did not research on human or animal subject.

## Supporting information


Figure S1
Figure S2
Figure S3
Figure S4
Figure S5
Figure S6

Table S1

## Data Availability

The data that support the findings of this study are openly available in GEO at https://www.ncbi.nlm.nih.gov/geo/query/acc.cgi?acc=GSE206999, reference number GSE 206999.
