# Supplementary material for: ESE1/AGR2 axis antagonizes TGF‐β‐induced epithelial‐mesenchymal transition in low‐grade pancreatic cancer
Source: Cancer Med. 2022 Nov 3;12(5):5979–93. doi: 10.1002/cam4.5397 (PMC10028153; doi:10.1002/cam4.5397)
Supplement: Supplementary file 1 — Figure S1 Figure S2 Figure S3 Figure S4 Figure S5 Figure S6 [file CAM4-12-5979-s001.docx]

**Supplementary Figure 1. ESE1 is overexpressed in various human cancer tissues including PDAC.**

(A) The ESE1 gene expression profile across different tumor samples and paired normal tissue. (B) The correlation between ESE1 expression and overall survival (OS) in PDAC patients based on the data obtained and analyzed from Kaplan-Meier Plotter database. COAD, Colon adenocarcinoma. READ, Rectum adenocarcinoma. PDAC, Pancreatic adenocarcinoma. BLCA, Bladder Urothelial Carcinoma. CESC, Cervical squamous cell carcinoma and endocervical adenocarcinoma. LUAD, Lung adenocarcinoma. KIRP, Kidney renal papillary cell carcinoma. STAD, Stomach adenocarcinoma. CHOL, Cholangiocarcinoma. ESCA, Esophageal carcinoma. UCEC, Uterine Corpus Endometrial Carcinoma. OV, Ovarian serous cystadenocarcinoma. LUSC, Lung squamous cell carcinoma. BRCA, Breast invasive carcinoma. PRAD, Prostate adenocarcinoma. HNSC, Head and Neck squamous cell carcinoma. LIHC, Liver hepatocellular carcinoma. KIRC, Kidney renal clear cell carcinoma. UCS, Uterine Carcinosarcoma. KICH, Kidney Chromophobe.


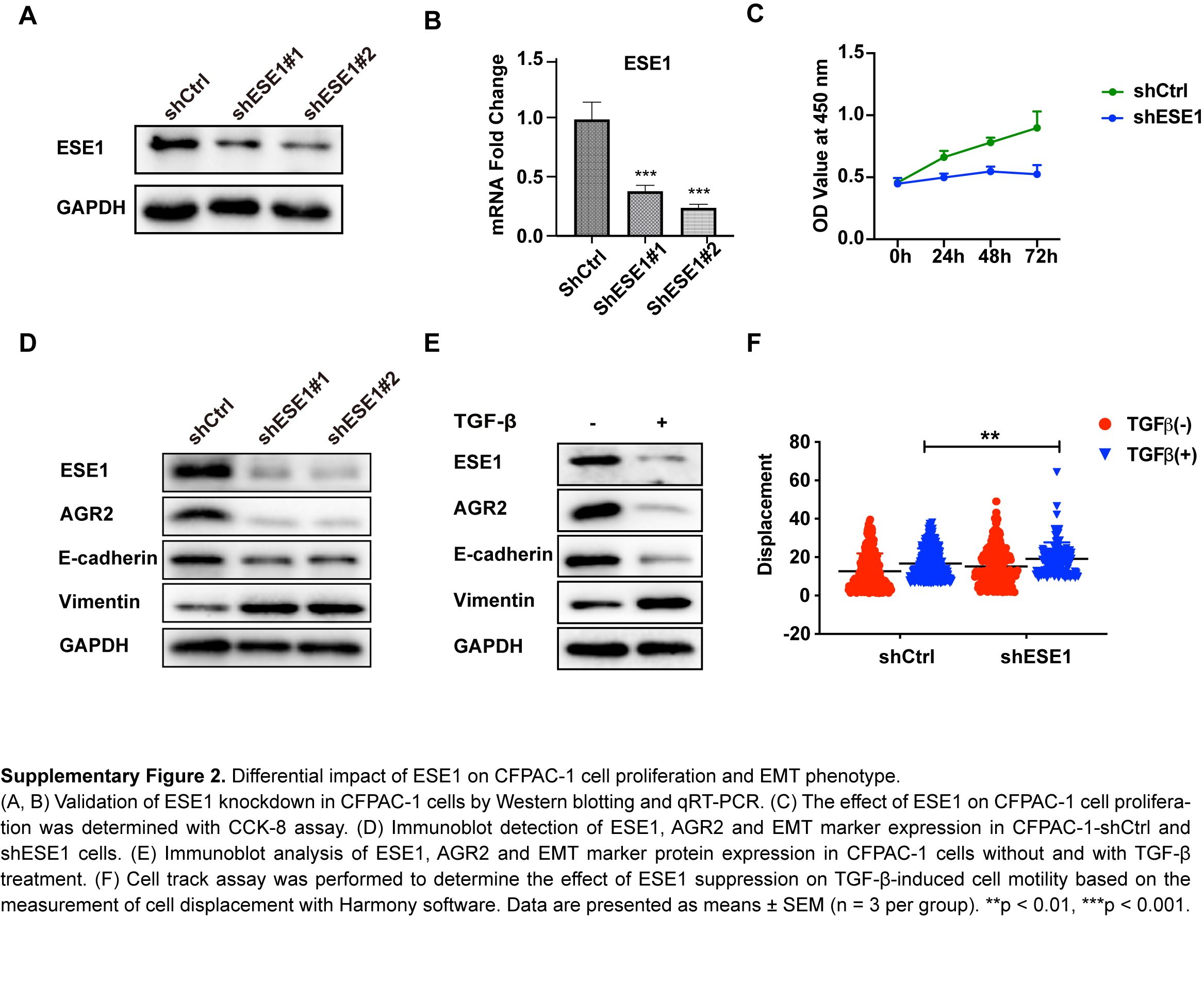


**Supplementary Figure 2.** Differential impact of ESE1 on CFPAC-1 cell proliferation and EMT phenotype.

(A, B) Validation of ESE1 knockdown in CFPAC-1 cells by Western blotting and qRT-PCR. (C) The effect of ESE1 on CFPAC-1 cell proliferation was determined with CCK-8 assay. (D) Immunoblot detection of ESE1, AGR2 and EMT marker expression in shCtrl and shESE1 CFPAC-1 cells. (E) Immunoblot analysis of ESE1, AGR2 and EMT marker protein expression in CFPAC-1 cells without and with TGF-β treatment. (F) Cell track assay was performed to determine the effect of ESE1 suppression on TGF-β-induced cell motility based on the measurement of cell displacement with Harmony software. Data are presented as means ± SEM (n = 3 per group). **p < 0.01, ***p < 0.001.

**Supplementary Figure 3.** Enhanced vimentin expression and EMT morphology in shESE1 L3.6 cells treated with TGF-β. Vimentin (red), nuclei (DAPI). Scale bars: 50μm.

**Supplementary Figure 4.** **AGR2 is overexpressed in human PDAC.** (A) The expression of AGR2 in primary PDAC and normal tissues based on analysis of TCGA database. (B) The expression of AGR2 in PDAC tissues based on individual cancer stage from the TCGA database. (C) The correlation between AGR2 expression with RFS in stage-2 PDAC based on Kaplan-Meier Plotter analysis. Validation of AGR2 knockdown efficiency by qRT-PCR (D), and western blotting (E). Representative images of colony formation assay (F), and wound-healing assay (G) from shCtrl and shAGR2 groups. (H) Immunoblot detection of EMT marker expression in shAGR2 L3.6 cells.

**Supplementary Figure 5.** **Correlation of ESE1 and AGR2 expression PDAC cells.**

(A) L3.6 cells transfected with a GFP-tagged lentiviral shRNA vector were stained for endogenous ESE1 and AGR2 by IF. GFP (green), ESE1(white), AGR2 (red), nuclei (DAPI). (B) Detection of endogenous ESE1 and AGR2 in CFPAC-1 cell by IF. ESE1(green), AGR2 (red), nuclei (DAPI). Scale bars: 25μm.

**Supplementary Figure 6.** **Luciferase reporter activity of ESE1 C-terminal truncation mutants in PDAC cells.** (A) Domain representation of full-length ESE1 and its truncated mutants. (B) The relative activities of indicated ESE1 truncation mutants based on the heterologous GAL4-Luciferase reporter assay in different PDAC cell lines. Data are presented as means ± SEM (n = 3 per group). ****p < 0.0001 compared to GAL4 DBD ctrl. *PNT, TAD, SAR, A/T* and *ETS* indicate Point domain, transactivation domain, serine and aspartic acid-rich domain, AT-hook domain, and ETS DNA binding domain, respectively. GAL4-Luc, 5x GAL4 site luciferase­­ reporter.
