# Supplementary material for: ESE1/AGR2 axis antagonizes TGF‐β‐induced epithelial‐mesenchymal transition in low‐grade pancreatic cancer
Source: Cancer Med. 2022 Nov 3;12(5):5979–93. doi: 10.1002/cam4.5397 (PMC10028153; doi:10.1002/cam4.5397)
Supplement: Supplementary file 2 — Table S1 [file CAM4-12-5979-s002.docx]

**Supplementary Table 1.**

Guide sequences for lentiviral shRNA expression

| shRNA name | shRNA guide sequence |
| --- | --- |
| shESE1(UTR) | 5'-AGATGTACATAGAGATCTATT-3' |
| shESE1(CDS) | 5'-GCCATGAGGTACTACTACAAAC-3' |
| shAGR2(CDS) | 5'-GACAAACACCTTTCTCCTGAT-3' |
| shAGR2(UTR) | 5'-CCTTGAGACTTGAAACCAGAA-3' |

CDS, coding sequence; UTR, an untranslated region

Primer sequences for qRT-PCR analysis

| Primer name | Primer sequences |
| --- | --- |
| ESE1-F | 5'-GGCCGATGACTTGGTACTGAC-3' |
| ESE1-R | 5'-GCTTGCGTCGTACTTGTTCTTC-3' |
| AGR2-F | 5'-GTCAGCATTCTTGCTCCTTG-3' |
| AGR2-R | 5'-GGGTCGAGAGTCCTTTGTGTC-3' |
| GAPDH-F | 5'-ACATCGCTCAGACACCATG -3' |
| GAPDH-R | 5'-TGTAGTTGAGGTCAATGAAGGG-3' |
| E‐cadherin-F | 5'-AATGCCGCCATCGCTTAC-3' |
| E‐cadherin-R | 5'-ACCAGGGTATACGTAGGGAAATCT-3' |
| Vimentin-F | 5'-GAGGATCTGGAATTCGGATCC‐3' |
| Vimentin-R | 5'-ACGCGTCGACTTATTCAAGGT-3' |

F, forward primer; R, reverse primer
